# Factors associated with tocolytic hospitalizations in Taiwan: evidence from a population-based and longitudinal study from 1997 to 2004

**DOI:** 10.1186/1471-2393-9-59

**Published:** 2009-12-18

**Authors:** Ke-Zong Michelle Ma, Edward C Norton, Eing-Mei Tsai, Shoou-Yih Daniel Lee

**Affiliations:** 1 Graduate Institute of Healthcare Administration, Kaohsiung Medical University, Kaohsiung, Taiwan; 2 Department of Health Management and Policy, University of Michigan, Ann Arbor, MI, USA; 3 Department of Economics, University of Michigan, Ann Arbor, MI, USA; 4 Department of Obstetrics and Gynecology, Graduate Institute of Medicine, Kaohsiung Medical University, Kaohsiung, Taiwan; 5 Kaohsiung Medical University Hospital, Kaohsiung, Taiwan; 6 Department of Health Policy and Management, University of North Carolina at Chapel Hill, Chapel Hill, NC, USA

## Abstract

**Background:**

The use of tocolytic hospitalization in antenatal care is controversial and worthy of more research. We investigated individual, institutional, and area factors that affect the use of tocolytic hospitalizations in Taiwan where fertility has rapidly declined.

**Methods:**

Longitudinal data from the 1996 to 2004 National Health Insurance Research Database in Taiwan were used to identify tocolytic hospitalizations. The probit model was used to estimate factors associated with tocolytic hospitalizations.

**Results:**

The decline in fertility was significantly associated with the probability of tocolytic hospitalizations. Several physician and institutional factors-including physician's age, hospital ownership, accreditation status, bed size, and teaching status-were also significantly correlated to the dependent variables.

**Conclusions:**

The provision of inpatient tocolysis is influenced not only by clinical considerations but also by physician, institutional, and area factors unrelated to clinical need. Fertility declines in Taiwan may have led obstetricians/gynecologists to provide more tocolysis to make up for their lost income. If the explanation is further validated, reimbursement policies may need to be reviewed to correct for overuse of inpatient tocolysis. The correlation could also be explained by the increasing use of artificial reproductive technologies and higher social value of newborns. In addition, the physician and institutional variations observed in the study indicate potential misuse of inpatient tocolysis that warrant further investigation.

## Background

Antenatal care generally improves maternal and infant health [1]. The most commonly stated reason for antenatal hospitalizations is having symptoms of threatened preterm labor [2-8]. Tocolytic treatment, which uses pharmacologic agents to inhibit uterine contractions and to prevent delivery before the completion of 37 weeks of gestation, is touted to reduce perinatal morbidity and mortality associated with threatened preterm labor [9,10]. However, the use of tocolytic hospitalization in antenatal care is controversial due to the potential adverse health effects and conflicting evidence of effectiveness [8,11-15]. Research indicated that antenatal hospitalizations with a pregnancy-related diagnosis may impose significant economic and psychosocial burdens on pregnant women and their family and may increase costs for the health care system [3,13,16]. Several U.S. studies showed that tocolytic treatment accounts for one-quarter to nearly one-half of all antenatal hospitalizations [3,5-7,17]. Examining the conditions under which women receive tocolytic treatment, including factors unrelated to the woman's clinical need, is necessary to understand whether the treatment are always justified.

Several studies have attempted to identify factors that lead to tocolytic hospitalizations. Most of these studies have focused on individual factors, such as pregnancy complications [3], adverse reproductive health history [5,14,18], severe life events [18], age [3-7], race [3,4,18], and insurance status [3,4,18]. By focusing on these individual variables, existing studies assume that the decision to provide antenatal and tocolytic treatments is predominantly a clinical consideration, unrelated to organizational and health care market conditions that have been found in numerous studies to affect medical service use [19-21]. Moreover, the majority of existing research uses either a cross-sectional study design or data from a selected patient subpopulation. It can be argued that a change in market conditions may encourage obstetricians and gynecologists to admit more women for tocolytic treatments for reasons unrelated to clinical need. This argument is testable when there is sufficient temporal variation in market conditions.

The purpose of this study is to conduct a longitudinal and population-based analysis of individual, organizational, and market factors that influence tocolytic hospitalizations. Data for this study were collected in Taiwan between 1996 and 2004. Two recent developments in Taiwan make the study particularly interesting. First, Taiwan has experienced a fertility decline since 1984 and the decline has been more dramatic in the last ten years. The number of live births in Taiwan was 325,545 in 1996, and it decreased to 204,414 in 2007 [22], giving Taiwan one of the lowest fertility rates among developed countries [23]. The fertility decline may have created an incentive for obstetricians and gynecologists to provide more tocolytic treatments to make up for lost revenue due to fewer births. The rapid fertility decline and its potential impact on tocolytic hospitalizations should be considered in empirical specifications.

Second, in March 1995, Taiwan implemented a new National Health Insurance program that provides health insurance coverage to the entire population. Currently, more than 95 percent of hospitals and clinics are contracted to provide health care services in the NHI program [24]. Physicians participating in the National Health Insurance program are paid according to fixed rates set by the Bureau of National Health Insurance, and the contracted medical institutions are paid mainly on a fee-for-service basis. Universal health insurance in Taiwan and the single-payer design offer a favorable research setting that prevents the use of cumbersome methods to control for variation and change in health insurance coverage often seen in countries, such as the U.S., that have fragmented health care systems.

## Methods

We employed a longitudinal design and used data from the National Health Insurance Research Database, which is maintained by the National Health Research Institute. Specifically, information from the National Health Insurance Research Database from 1996 to 2004 was combined into a single file that contained discharge records of National Health Insurance enrollees: registry for contracted medical facilities, registry for medical personnel, registry for contracted beds, registry for beneficiaries, registry for board-certified specialists, hospital discharge file, and registry for catastrophic illness patients. We were able to link discharge data with hospital and provider information because each discharge record contained patient, hospital and provider IDs. Further, we obtained data on the general fertility rate and population size from the Taiwan-Fuchien Demographic Fact Book, 1997-2004, and merged them with the National Health Insurance Research Database by hospitals' area codes.

To ensure confidentiality, all IDs were scrambled. Any information that could be used to identify individual enrollees was also deleted before the data were released to the researchers. Because the risk of identifying individuals from the released data is minimal, the National Health Research Institute approved our use of the National Health Insurance Research Database and the institutional review boards at the University of North Carolina at Chapel Hill and the Kaohsiung Medical University determined that the study did not constitute human subjects research and granted a waiver.

The study population included women who gave birth between January 1, 1997 and December 31, 2004. To be consistent with previous research, the following exclusion criteria were applied: (1) women hospitalized for early pregnancy loss (ICD-9-CM 630-634 and 637-639) or elective termination (ICD-9-CM 635 and 636); (2) women aged above 50 or below 15; (3) women whose attending obstetrician/gynecologist's age was below 25 or above 75; and (4) women with multiple deliveries. Pregnancies in women under 15 or over 50 years old are atypical. Clinicians in Taiwan, as elsewhere, are rarely qualified as obstetricians/gynecologists before the age of twenty-five or after seventy-five. Women with multiple pregnancies may be particularly prone to antenatal hospitalizations [25].

Because our focus was on tocolytic hospitalizations, defined as ICD-9-CM codes 644.0 to 644.4 listed in the diagnosis field, women contraindicated for tocolysis according to the current standard of care and women noted to have additional medical conditions that could have been treated with medications misclassified with tocolysis were also excluded [26]. Those exclusion conditions included hypertension/eclampsia/pre-eclampsia (ICD-9-CM 642), excessive maternal bleeding/abrupted placenta/placenta previa (ICD-9-CM 762.0, 762.1, 762.2), premature rupture of membrances/incompetent cervix (ICD-9-CM 761), fetal distress (ICD-9-CM 656.3, 663.0, 768.3 and 768.4), maternal infection/chorioamnionitis (ICD-9-CM 762.7), and congenital abnormalities (ICD-9-CM 740-759). Based on the above inclusion and exclusion criteria, the final sample of our study contained 83,619 observations of tocolytic hospitalization. Totally, 64,098 cases were excluded in our study.

The unit of analysis was a pregnancy, and the dependent variable was whether a tocolytic hospitalization occurred. The independent variables fell into four categories: individual, physician, institutional, and area factors. Individual factors included the woman's age, wage, having prior pregnancy-associated hospitalizations (ICD-9-CM codes from 640 to 676 with a fifth digit of "0" or "3", or any diagnosis in combination with a code V22 [normal pregnancy] or V23 [high-risk pregnancy]), having a major disease card (an indicator of having a severe health problem such as malignant neoplasm, end-stage renal disease, chronic psychotic disorder, cirrhosis of the liver, acquired immunodeficiency syndrome, and schizophrenia), and previous year's inpatient expenses. Age, having a major disease card, having prior pregnancy-associated hospitalizations, and previous year's inpatient expenses captured the health conditions of pregnant women that increased the likelihood of tocolytic hospitalization. Our analysis was focused on the use of tocolytic hospitalizations (ICD-9-CM codes from 644.0 to 644.4), which are a subset of antenatal hospitalizations (ICD-9-CM codes from 640 to 676 with a fifth digit of "0" or "3", or any diagnosis in combination with a code V22 [normal pregnancy] or V23 [high-risk pregnancy]).

Physician characteristics included attending obstetrician/gynecologist's age and gender. The attending obstetrician/gynecologist's years in the specialty was not included because it was highly correlated with age [27,28]. Institutional factors included hospital ownership (public, private non-profit, or proprietary), teaching status (teaching versus non-teaching institution), accreditation status (medical center, regional hospital, district hospital, or obstetric/gynecological clinic), and number of beds. In Taiwan, obstetric/gynecological clinics are allowed to have up to nine beds for inpatient services and can provide inpatient tocolytic treatment. An area factor, the annual general fertility rate in the region, was included. The general fertility rate was age-adjusted. Geographic areas in Taiwan are delineated based upon the health care service regions reported by the Department of Health in Taiwan. There are twenty-one regions. Finally, we included a full set of regional and time dummies to control for the regional and time fixed effects.

Because the dependent variable (having a tocolytic hospitalization) was binary, we estimated a probit model for this outcome. The probit model is a popular specification for a binary-response model that emerges from the normal cumulative distribution function (CDF). The probit model on the full sample was to predict the probability of a tocolytic hospitalization. In this model the probability will lie between 0 and 1 and vary nonlinearly with the explanatory variables. These properties are in contrast with the linear probability model where the probability increases linearly with the explanatory variables and may lie outside the 0-1 range.

The probit model can be written as:

where *Y *is a dichotomous variable indicating whether a mother had a tocolyic hospitalization (0 if no, 1 if yes), Φ is the standard normal cumulative distribution, *i *indexes individual patient, *g *indexes the obstetrician/gynecologist, *h *indexes the hospital, *r *indexes the region, *t *indexes time, the vector *W *represents all exogenous explanatory variables, *γ *is a vector of coefficients on *W*, and *ν *is the random error assumed to be independent of all other error terms.

Specifically, *W *= (*X*, *Z*, *H*, *ln*(*Fertility_rt_*), *δ_r_*, *ς_t_*), where, *ln*(*Fertility_rt_*) is the natural logarithm of regional age-adjusted fertility rate in year *t*, *δ_r _*is a set of dummies representing regions, *ς_t _*is a full set of year dummies, *X *is a vector of observable patient characteristics, *Z *is a vector of observable physician characteristic, and *H *is a vector of observable hospital characteristics. The regional fixed effects (*δ_r_*) would capture unobserved regional preferences for care or hospital selection.

The estimated parameters in probit model are not are not directly interpretable, but they can be translated into marginal effects. The magnitude of the marginal effect represents a percentage point change in the probability as a function of a change in a certain explanatory variable while keeping all the other covariates constant. Empirically, we use the "average of the probabilities" method to calculate the marginal effects of each covariate.

## Results

Table 1 contains summary statistics of the number of singleton deliveries, antenatal hospitalizations, and tocolytic hospitalizations from 1997 to 2004. Among the 1,979,311 women who gave birth during the study period, 83,619 experienced tocolytic hospitalizations. Notably, the use of tocolytic hospitalizations was not parallel to the decreasing number of newborns in recent years in Taiwan. The rates of inpatient antenatal hospitalizations, and tocolytic hospitalizations were 5.24% and 4.22%, lower than those reported in the literature, despite the use of similar inclusion and exclusion criteria in our sample selection [3,4,7,8]. It is possible that the coverage of ten prenatal outpatient visits in Taiwan's Nation Health Insurance may allow early detections of pregnancy-related problems and reduce the need for tocolytic hospitalizations.

**Table 1:** Summary statistics of singleton deliveries and antenatal hospitalizations, 1997-2004

Year	Number of Singleton deliveries	Total number of antenatal hospitalizations	Total number of tocolytic hospitalizations	Number of excluded cases
1997	302,049	13,341	9,145	8,814
1998	246,244	12,236	9,816	8,262
1999	257,326	12,441	10,217	9,025
2000	276,786	13,311	11,567	8,890
2001	239,626	13,205	10,682	7,644
2002	232,192	13,149	10,862	7,495
2003	216,390	13,310	10,732	6,609
2004	208,698	12,318	10,598	7,359

Total	1,979,311	103,811	83,619	64,098

The exclusion population include: (1) women hospitalized for early pregnancy loss (ICD-9-CM 630-634 and 637-639) or elective termination (ICD-9-CM 635 and 636); (2) women aged above 50 or below 15; (3) women whose attending obstetrician/gynecologist's age was below 25 or above 75; (4) women with multiple deliveries (ICD-9-CM 651-652); (5) hypertension/eclampsia/pre-eclampsia (ICD-9-CM 642); (6) excessive maternal bleeding/abrupted placenta/placenta previa (ICD-9-CM 762.0, 762.1, 762.2); (7) premature rupture of membrances/incompetent cervix (ICD-9-CM 761); (8) fetal distress (ICD-9-CM 656.3, 663.0, 768.3 and 768.4) (9) maternal infection/chorioamnionitis (ICD-9-CM 762.7); and (10) congenital abnormalities (ICD-9-CM 740-759).

The descriptive statistics in Table 2 indicate that women experiencing tocolytic hospitalizations were generally older (29.1 vs. 28.2 years old), had poorer health status in terms of having a major disease card (2.1% vs. 1.3%), had prior pregnancy-associated hospitalizations (38.2% vs. 3.6%), and had higher inpatient expenses in the previous year (NT$3,968.9 vs. NT$3,637.2), compared to all women who gave birth. Women experiencing tocolytic hospitalizations also had higher wages (NT$18,393.5 vs. NT$17,202.9). The last column of Table 2 showed the descriptive statistics about the excluded population, and they were also older and unhealthier compared to the general population.

**Table 2:** Summary statistics of the study population

Variables	Women who gave birth	Women who gave birth and had a tocolytic hospitalization	Excluded population
***Individual factors***			
Age (S.D.)	28.22 (4.9)	29.12 (5.3)	29.27 (5.9)
Wage (S.D.)	17,202.93 (16,325.7)	18,393.48 (17,256.6)	18,215.43 (16,957.2)
Having a major disease card (%)	25,719 (1.3%)	1,733 (2.1%)	2,006 (1.9%)
Having hospitalizations for preterm labor before (%)	71,876 (3.6%)	31,935 (38.2%)	18,897 (18.2%)
Previous year's inpatient expenses (S.D.)	3,637.16 (14198.1)	3,968.92 (14444.2)	3,789.52 (14264.86)

Observations	1,979,311	83,619	64,098

Results on the use of tocolytic hospitalizations are reported in Table 3. All estimates are reported with robust standard errors to fix the heteroskedasticity problem in probit model. According to the probit model, several explanatory variables such as women's age, wage, having prior pregnancy-associated hospitalizations, previous year's inpatient expenses, hospital bed size, public hospital ownership, hospital accreditation status (non-clinic), teaching hospital, attending obstetrician/gynecologist's age displayed a positive and statistically significant association with the probability of having a tocolytic hospitalization. Private non-profit hospital ownership was negatively correlated with the propensity of having a tocolytic hospitalization. Interestingly, regional fertility rate was negatively associated with tocolytic hospitalization, indicating that a decrease in the regional fertility rate was significantly correlated with an increase in the propensity of tocolytic hospitalization. In terms of marginal effects, a one percent decrease in the regional fertility rate was associated with a 0.221 percentage points increase on average in the probability of having a tocolytic hospitalization. The interpretations of marginal effects of other explanatory variables are similar, e.g., teaching hospitals are associated with a 0.05 percentage points increase on average in the probability of having a tocolytic hospitalization than non-teaching hospitals.

**Table 3:** Probit model for the use of tocolytic hospitalizations

Variables	Probit model	Marginal effect
Constant term	-3.028 (0.423)**	-2.326 (0.074)**
***Individual factors***		
Age	0.026 (0.001)**	-0.010 (0.0002)**
Wage	0.004 (0.0001)**	0.001 (0.0001)**
Having a major disease card	0.015 (0.018)	0.061 (0.065)
Having pregnancy-associated hospitalizations before	0.428 (0.006)**	0.665 (0.018)**
Previous year's inpatient expenses	0.001 (0.0002)**	0.027 (0.001)**
***Institutional factors***		
Bed size	0.004 (0.001)**	0.007 (0.001)**
Ownership		
Public	0.059 (0.010)**	-0.020 (0.004)**
Private non-profit	-0.102 (0.010)**	-0.042 (0.004)**
Proprietary (Reference)		
Accreditation status		
Medical center	0.392 (0.021)**	0.013 (0.007)**
Regional hospital	0.102 (0.017)**	0.021 (0.006)**
District hospital	0.113 (0.013)**	0.015 (0.005)**
Clinic (Reference)		
Teaching status		
Teaching	0.128 (0.011)**	0.050 (0.004)**
Non-teaching (Reference)		
***Physician factors***		
Male gender	-0.012 (0.010)	-0.004 (0.002)
Age	0.003 (0.0005)**	0.019 (0.001)**
***Area factor***		
Logged regional fertility rate	-0.539 (0.112)**	-0.221 (0.042)**

Observations	1,979,311	1,979,311

Log-likelihood	-148,762.7	

Robust standard errors are in parentheses. The three models also include a full set of time and regional dummies. *Statistically significant at the 10% level. **Statistically significant at the 5% level.

## Discussion

Our study provides a comprehensive analysis of tocolytic hospitalizations in Taiwan. The validity of our study may be affected by the inherent limitations of the administrative data (e.g., limited diagnostic and clinical information, reporting errors) and the lack of detailed patient-level data such as gestational age [29], parity, pre-pregnancy body mass index, gestational weight gain [30,31], neonatal outcomes (e.g., prematurity and extreme prematurity), tocolytic agents (e.g., ritodrine and nifedipine), and antenatal steroids (e.g., glucocorticoids). Our findings may have limited generality because the data were somewhat dated and because of the particular medical practice and health insurance situations in Taiwan. Despite these caveats, several of our findings are noteworthy and may have implications for countries that experience a similar decline in fertility.

An important finding is that declining fertility was associated with increased use of tocolytic hospitalizations. There are several explanations for this finding. The first is the financial incentive. In Taiwan, obstetricians/gynecologists either are employed by hospitals (on hospital payroll) or operate their own clinics. The salary of those employed by hospitals usually constitutes two components: the contracted annual salary and a bonus based on the volume and level of services they provide. The income of self-employed obstetricians/gynecologists is affected by the volume of services they provide. As fertility decline reduces the number of deliveries-an important revenue source-obstetricians/gynecologists as well as hospitals may have an incentive under the current fee-for-service payment structure to provide more inpatient tocolysis services in order to maintain, or increase, their revenue. Patients are unlikely to object, because they bear no financial risk under the National Health Insurance regime and because they lack the knowledge to assess the medical risk of tocolysis. To the extent this explanation is valid, our study offers a precautionary note to countries that have experienced a fertility decline and where the health care payment system similarly presents a financial incentive for obstetricians/gynecologists and hospitals to offer unwarranted services.

Second, tocolytic treatments may be related to the conditions accompanying the declining fertility rate in Taiwan, including late marriage, older childbearing age, and increased use of artificial reproductive technologies and services. Use of artificial reproductive technologies is known to increase the likelihood of preterm birth, multiple-gestation pregnancies, and other high-risk conditions [32,33]. Lower fertility and getting pregnant later in life may increase the use of artificial reproductive technologies, which in turn may increase inpatient tocolytic treatment. Recent statistics by the Bureau of Health Promotion indicate that the use of artificial reproductive technologies has increased slightly from 1998 to 2006 [34]. We were unable to control for the use of artificial reproductive technologies in our analysis because such use is not covered by Taiwanese National Health Insurance and the utilization data are unavailable in the National Health Insurance claims file. Research is needed to explore the relationship between use of artificial reproductive technologies and tocolytic treatment and the findings could have both clinical and policy implications.

A third explanation is that pregnant women may request tocolytic treatment on the assumption that the treatment ensures a better birth outcome. A high premium is placed on babies as a result of decreased fertility and fewer children per family. Thus, the relationship between low fertility and high use of tocolytic hospitalizations possibly reflects a higher social value of newborns.

Some of these explanations suggest potential misuse of tocolytic hospitalizations and would warrant further investigation. For example, if increased use of tocolytic hospitalizations is largely explained by provider financial incentives, then reimbursement policies should be adjusted to prevent unnecessary use. Alternatively, if tocolytic hospitalizations are made primarily by maternal requests, a public education campaign may be effective in reducing overuse.

Also interesting are our findings that tocolytic hospitalizations were influenced by not only clinical factors but also physician and institutional conditions that had little relevance to clinical considerations. A possible explanation is that high-risk deliveries may have much better outcomes when they are transferred to a tertiary-level hospital with a high volume of obstetric and neonatal services [35]. Therefore, tertiary-level hospitals are likely to provide more tocolytic hospitalizations. On the other hand, such physician and institutional variations are noteworthy in light of evidence that for women with preterm labor, tocolysis may be unnecessary, often ineffective, and occasionally harmful [9,13,14,36,37]. Is it possible that ineffective and poor outcomes of inpatient tocolytic treatments are associated with the physician and institutional variations observed in this study? If so, variations in tocolytic hospitalizations raise access and cost concerns and have important quality implications.

An important premise of the study is that effective strategies for rational management of tocolytic hospitalizations require a comprehensive understanding of factors affecting inpatient maternal services. To reduce medically unnecessary tocolytic hospitalizations, reimbursement policy may be the tool of choice for policymakers. For example, under the current fee-for-service payment scheme in Taiwan, reimbursement based on a well-designed diagnosis-related grouping system may lead to more appropriate use of tocolytic treatments. In addition, continuing education for health care professionals may be an important tool to reduce unnecessary tocolysis. Other options to constrain overutilization include the application of technology (e.g., the use of both the measurement of cervicovaginal fetal fibronectin and transvaginal sonographic assessment of cervical length [38]) and peer-review of individual cases. Because evidence remains ambiguous about the effectiveness of inpatient tocolysis, obstetricians/gynecologists and health services researchers should also explore the costs and effectiveness of inpatient and outpatient tocolysis, using more detailed clinical data and well-controlled study design to provide more convincing empirical evidence. Future research could also assess the relationship between tocolytic care and consequential maternal as well as neonatal health outcomes.

## Conclusion

Overall, our study shows that the provision of inpatient tocolysis is influenced not only by clinical considerations but also by physician, institutional, and area factors unrelated to clinical need. Physician financial incentive, increasing use of artificial reproductive technologies, and higher social value of newborns may explain the correlation between declining fertility and rate on the use of inpatient tocolysis. Physician and institutional variations observed in the study indicate potential misuse of inpatient tocolysis that warrant further investigation.

## Competing interests

The authors declare that they have no competing interests.

## Authors' contributions

KM proposed the study, participated in data preparation and analyses, and drafted the manuscript. ECN and SDL participated in the study design and helped to draft the manuscript. ET provided clinical expertise and critical review of the manuscript. All authors read and approved the final manuscript.

## Pre-publication history

The pre-publication history for this paper can be accessed here:

http://www.biomedcentral.com/1471-2393/9/59/prepub
